# Energy expenditure computation of a single bursting neuron

**DOI:** 10.1007/s11571-018-9503-3

**Published:** 2018-09-03

**Authors:** Fengyun Zhu, Rubin Wang, Xiaochuan Pan, Zhenyu Zhu

**Affiliations:** 1 School of Computer Science, Hangzhou Dianzi University, Hangzhou, China; 2 Institute of Cognitive Neurodynamics, East China University of Science and Technology, Shanghai, 200237 China

**Keywords:** Bursting, Chay model, Neural energy, Na/K-ATPase pump, Energy coding

## Abstract

Brief bursts of high-frequency spikes are a common firing pattern of neurons. The cellular mechanisms of bursting and its biological significance remain a matter of debate. Focusing on the energy aspect, this paper proposes a neural energy calculation method based on the Chay model of bursting. The flow of ions across the membrane of the bursting neuron with or without current stimulation and its power which contributes to the change of the transmembrane electrical potential energy are analyzed here in detail. We find that during the depolarization of spikes in bursting this power becomes negative, which was also discovered in previous research with another energy model. We also find that the neuron’s energy consumption during bursting is minimal. Especially in the spontaneous state without stimulation, the total energy consumption (2.152 × 10^−7^ J) during 30 s of bursting is very similar to the biological energy consumption (2.468 × 10^−7^ J) during the generation of a single action potential, as shown in Wang et al. (Neural Plast 2017, 2017a). Our results suggest that this property of low energy consumption could simply be the consequence of the biophysics of generating bursts, which is consistent with the principle of energy minimization. Our results also imply that neural energy plays a critical role in neural coding, which opens a new avenue for research of a central challenge facing neuroscience today.

## Introduction

Understanding neural coding remains a central focus of neuroscience since the pioneering research of Edgar Adrian in the 1930s (Adrian 1932). The question is critical to elucidating the working mechanisms of the brain. A variety of proposed neural coding patterns, such as rate coding, time coding, phase coding and population coding (Optican and Richmond 1987; Lee et al. 1988; Igarashi et al. 2007; Cessac et al. 2010), are applicable only to local neural activity research, and have limited use for the exploration of global brain activity (Butts et al. 2007; Gong et al. 2010; Guo and Li 2012).

To overcome this limitation, researchers have developed an energy coding theory (Wang et al. 2006, 2014, 2015a; Wang and Wang 2014; Wang and Zhu 2016) based on the principle that neural activities are in fact energy-expensive coding processes (Hyder et al. 2013; Attwell and Laughlin 2001; Torrealdea et al. 2009; Alle et al. 2009). While this theory is still in the exploratory stage, the core idea of examining the quantitative relationship between neural activity and neural energy consumption may offer a major contribution in understanding neural coding (Wang et al. 2006, 2015b). Building on this theory, recent research has achieved important results with applying various neural models and energy calculation methods (Zheng and Wang 2012; Zheng et al. 2014, 2016, 2017a, b). (I) Using the concepts of minimum mutual information and maximum entropy to study neural coding shows that the neural information processing of the brain follows the principles of minimization of energy consumption and maximization of information transmission efficiency (Zheng and Wang 2012; Laughlin and Sejnowski 2003). (II) The calculations from the energy model in Zheng et al. (2014), demonstrate that during action potentials, neurons first release stored energy very rapidly and then receive from oxyhemoglobins the energy required for subsequent action potentials. (III) Wang et al. (2017a) based on the Hodgkin–Huxley model calculates by the method of ion counting and power integration that an action potential consumes 2.468 × 10^−7^ J of biological energy produced by the hydrolysis of Adenosine triphosphate (ATP). These results show that it is reasonable and effective to explore the mechanism of neural information processing by studying the relationship between neurons’ firing activity and the energy they consume.

To understand neural processing mechanisms we also need to study the dynamics of neuronal subthreshold electrical activities. Neuronal bursting, a common firing pattern of these electrical activities observed in many electrophysiological experiments, has been studied extensively (Ji et al. 2015; Izhikevich 2000; Kepecs and Wang 2000; Wang et al. 2011; Shi et al. 2017; Zhang et al. 2016; Perc and Marhl 2005; Gu et al. 2015; Jia et al. 2017; Duan et al. 2017). These studies mainly focus either on mathematical models for numerical analysis or on elucidating the time coding characteristics of bursting activities (Ji et al. 2015; Izhikevich 2000; Kepecs and Wang 2000; Wang et al. 2011; Shi et al. 2017). The latter approach is more common because it is assumed that the coding information is contained in the precise time structure between spikes or between bursts (Zhang et al. 2016; Perc and Marhl 2005; Gu et al. 2015; Jia et al. 2017; Duan et al. 2017). However, most such studies rely mainly on data calculation and cannot explain the biological significance of the bursting phenomenon. So far there is no consensus as to this significance (Krahe and Gabbiani 2004; Wang 1999; Li et al. 2016; Izhikevich et al. 2003; Li et al. 2009; Bera et al. 2017). One study argues that bursting can strengthen synaptic plasticity (Li et al. 2016). Another study posits that bursts can be used in the selective communication between neurons (Izhikevich et al. 2003). Still another study found in the case of sleeping rats that the bursting of a single cortical neuron could trigger shifts between the states of slow-wave sleep (SWS) and rapid eye movement (REM) (Li et al. 2009). In addition, bursting has also been related to chimera states in neuronal dynamics (Bera et al. 2017). Further study in this field, therefore, is obviously necessary to obtain a better understanding of the biological significance of bursting.

Starting from experimental observations that neural firing activity consumes energy, this study uses the Chay model to propose a method of calculating the energy consumption of a bursting neuron. Using these results, we study the influence of ion flows on changes in both membrane potential and its corresponding power and analyze the fluctuations of the total energy consumption induced by various electrical stimuli to obtain a deeper understanding of firing activity within the neural system.

## Methods

### Chay model

**Fig. 1:**
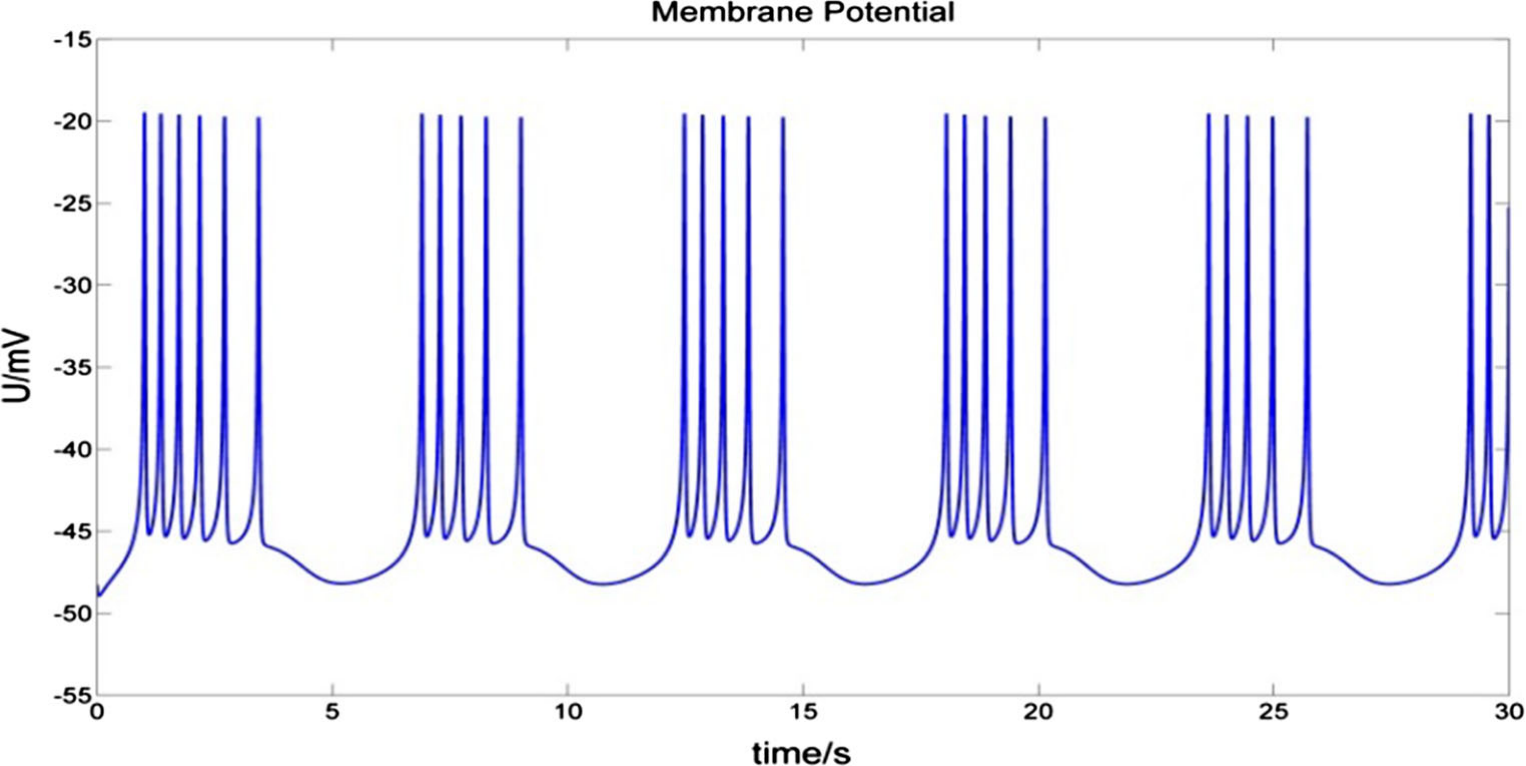
Burst firing simulated by the Chay model. The membrane potential varies between − 55 and − 15 mV, and each burst contains several spikes. *V*_i_ = 100 mV, *V*_k_ = − 75 mV, *V*_l_ = − 40 mV, *V*_c_ = 100 mV; g_i_ = 1800 nS, g_kv_ = 1700 nS, g_kc_ = 11.5 nS, g_l_ = 7 nS. For a detailed description of the Chay model please refer to Chay (1985)

The Chay model can simulate bursting activities of neurons of a biological nervous system in a simple yet effective way (Chay 1985; Chay and Fan 1995) (Fig. 1). This model contains the following ionic currents: inwards Na^+^–Ca^2+^ mixed channel ions, outwards voltage-dependent K^+^ channel ions, Ca^2+^-dependent K^+^ channel ions and leakage channel ions. The Chay model is described using the following three differential equations:

1 $$\begin{aligned} \frac{dV}{dt} & = g_{i} m_{\infty }^{3} h_{\infty } (V_{i} - V) + g_{kv} n^{4} (V_{k} - V) \\ & \quad + g_{kc} \frac{C}{1 + C}(V_{k} - V) + g_{l} (V_{l} - V) + I \\ \end{aligned}$$

2 $$\frac{dn}{dt} = \frac{{n_{\infty } - n}}{{\tau_{n} }}$$

3 $$\frac{dC}{dt} = \rho \left( {m_{\infty }^{3} h_{\infty } (V_{C} - V) - k_{C} C} \right)$$

$m_{\infty }$, $h_{\infty }$, $n_{\infty }$ and $\tau {}_{n}$ can be expressed by:

$$\begin{aligned} & y_{\infty } = \frac{{\alpha_{y} (V)}}{{\alpha_{y} (V) + \beta_{y} (V)}}\quad (y = m,h,n); \\ & \alpha_{m} (V) = 0.1(25 + V)/(1 - e^{ - 0.1V - 2.5} ), \\ & \beta_{m} (V) = 4e^{ - (V + 50)/18} ; \\ & \alpha_{h} (V) = 0.07e^{ - 0.05V - 2.5} , \\ & \beta_{h} (V) = 1/(1 + e^{ - 0.1V - 2} ); \\ & \alpha_{n} (V) = 0.01(20 + V)/(1 - e^{ - 0.1V - 2} ), \\ & \beta_{n} (V) = 0.125e^{ - (V + 30)/80} ; \\ & \tau_{n} = 1/(\lambda_{n} (\alpha_{n} + \beta_{n} )); \\ \end{aligned}$$

Here *V*, *n*, and *C* are the membrane potential, the probability of opening voltage-dependent K^+^ channels, and the intracellular Ca^2+^ concentration, respectively. *V*_i_, *V*_k_, *V*_c_ and *V*_l_ are the reversal potentials (which are also referred to as “Nernst potentials”) for mixed Na^+^–Ca^2+^, K^+^, Ca^2+^ and leakage ions, respectively. *g*_*i*_, *g*_*kv*_, *g*_*kc*_, and *g*_*l*_ represent the maximum conductance of the mixed Na^+^–Ca^2+^, voltage-dependent K^+^, Ca^2+^-dependent K^+^, and leakage ions respectively. $m_{\infty }$, $h_{\infty }$ are the probabilities of activation and inactivation of the mixed inward current channel, and $n_{\infty }$ is the steady-state value of n. $\tau {}_{n}$ is the relaxation time of the voltage-gated K^+^ channel. $k_{C}$ is the rate constant for the efflux of the intracellular Ca^2+^, and $\rho$ is a proportionality constant. *I* is the stimulus current.

### The energy calculation method

**Fig. 2:**
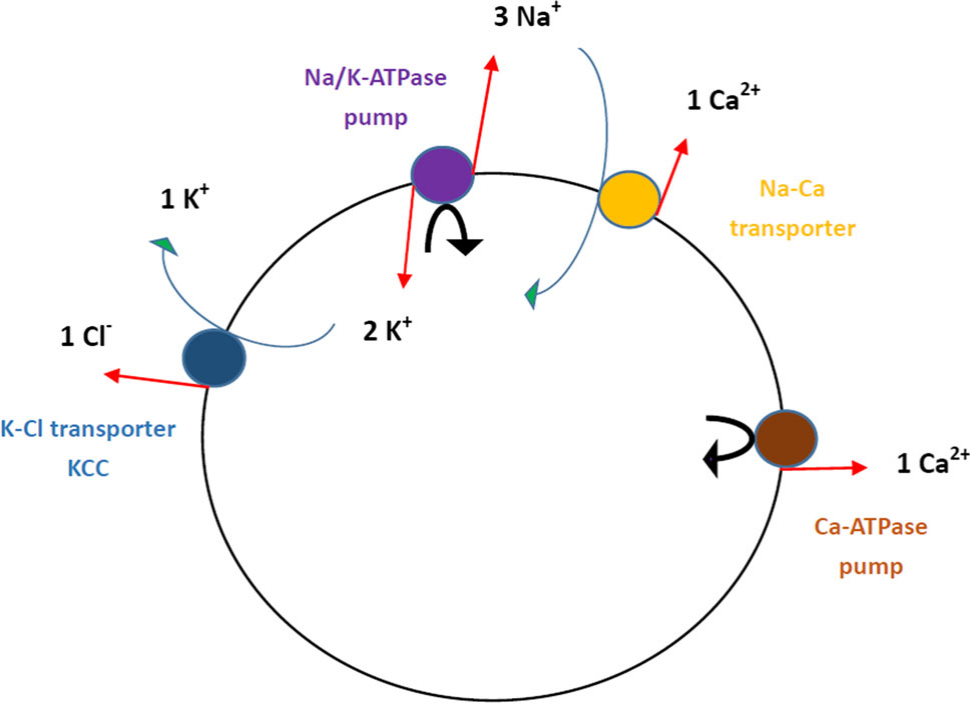
Schematic diagram of major ion pumps (Na/K-ATPase pump and Ca-ATPase pump) and tansporters (Na–Ca transporter and K–Cl tansporter) across the neuronal membrane. The red arrows represent the ion movement against the ion concentration gradient; the green arrows represent the ion movement along the ion concentration gradient; the black arrows represent the hydrolysis of biological energy ATP. These two pumps acquire energy directly from the hydrolysis of ATP to pump ions across the membrane against their electrochemical gradients. Other transporters do not consume ATP directly but rather take advantage of the electrochemical gradients of Na^+^ and K^+^ (or other ions), which ultimately depend on the hydrolysis of ATP by ATPase pumps, such as the Na/K-ATPase pump [Fig. 2 from Hammond (2008)]. (Color figure online)

During spontaneous firing, the difference in membrane potential depends on the selective permeability of the neuronal membrane and the ion concentration gradients across the membrane (Hyder et al. 2013; Gazzaniga et al. 2008; Hammond 2008). The selective permeability of membranes is due largely to ion channels, proteins that allow only certain kinds of ions to cross the membrane in the direction of their concentration gradients. As ions take their electrical charge with them as they go, electrical potential (which carries neural information) is generated. However, the activity of these ion channels relies on the establishment and maintenance of ion concentration gradients by ion pumps, which require biological energy ATP to pump ions across the neuronal membrane against ion concentration gradients (Gazzaniga et al. 2008; Hammond 2008), as the sodium–potassium pump (Na/K-ATPase pump) and calcium pump (Ca-ATPase pump) show in Fig. 2. The Na/K-ATPase pump actively extrudes three Na^+^ and imports two K^+^ for the hydrolysis of one ATP to generate and maintain transmembrane concentration gradients for Na^+^, K^+^ and indirectly for other ions.

**Fig. 3:**
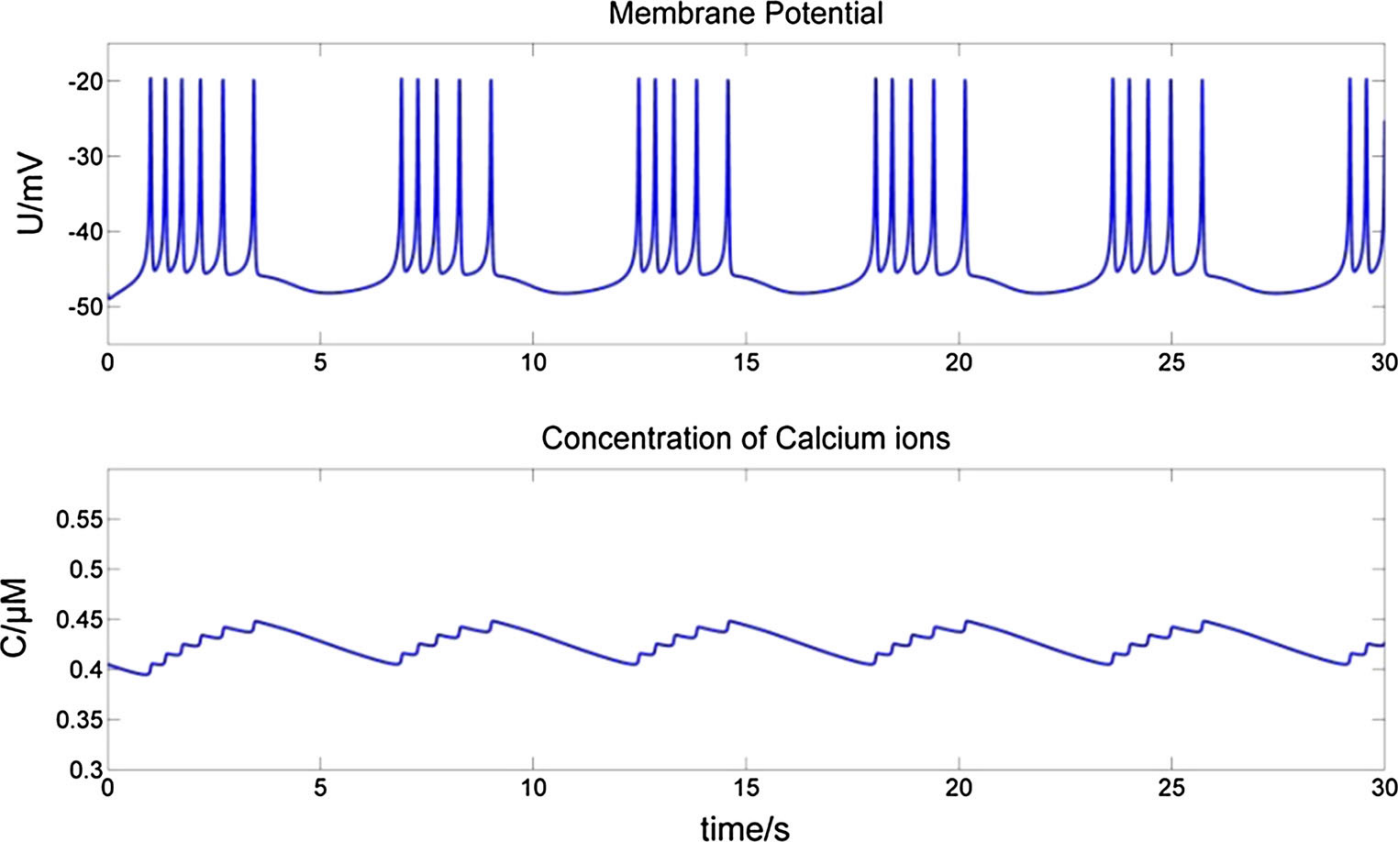
Changes of intracellular Ca^2+^ concentration during bursting. The intracellular Ca^2+^ concentration varies by about 0.05 µM and not more than 0.1 µM during burst firing

The neural energy calculated in this paper is only the biological energy ATP consumed by Na/K-ATPase pumps, which effectively contributes to electrical signals. It should be noted that the ATP consumed by the Ca-ATPase pump is ignored here for following two reasons. First, the intracellular Ca^2+^ concentration is very low compared to that of other ions. At rest, the neuron has a K^+^ concentration of 3 mM outside the membrane and 140 mM inside; a Na^+^ concentration of 140 mM outside and 7 mM inside; a Cl^−^ concentration of 40 mM outside and 7 mM inside; and a Ca^2+^ concentration of 1.5 mM outside and 0.1 µM inside (Hammond 2008). Second, the role of Ca^2+^ in this Chay model is considered in the mixed Na^+^–Ca^2+^ channel, with the concentration changes of intracellular Ca^2+^ varying by about 0.05 µM and not more than 0.1 µM (Fig. 3). In addition, the change of Ca^2+^ concentration depends mainly on Na–Ca transporters (Fig. 2) to pump Ca^2+^ out of neurons (0.5–1.0 µM) under the effect of the Na^+^ electrochemical gradient, which does not consume ATP, and much less on the Ca-ATPase pump (0.2–0.3 µM), which does consume ATP (Hammond 2008). This further indicates the rationality of ignoring the ATP consumption of the Ca-ATPase pump.

**Fig. 4:**
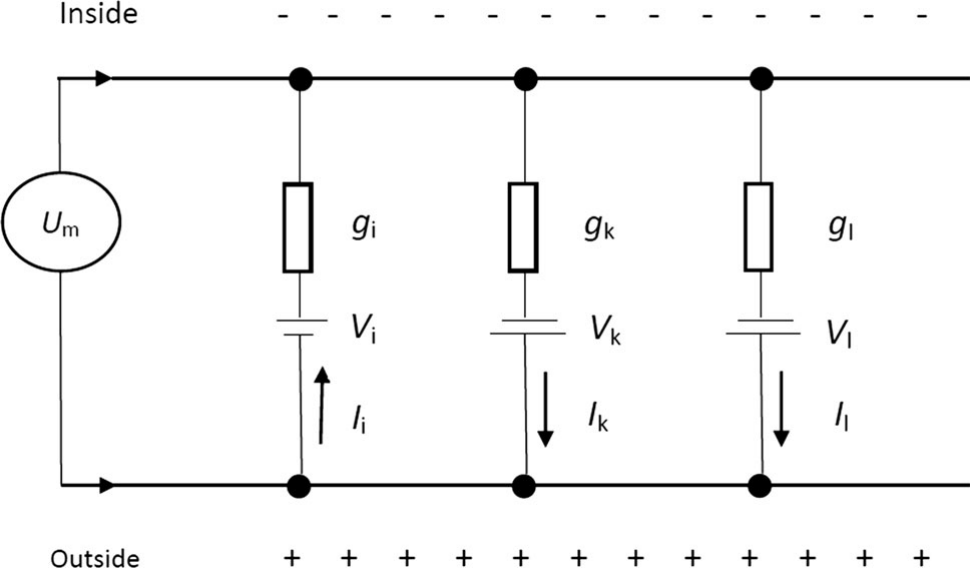
Schematic diagram of the electrical circuit of the neural membrane. Voltage sources (*V*_*i*_, *V*_*k*_, *V*_*l*_) in this circuit correspond to reversal potentials for mixed Na^+^–Ca^2+^, K^+^ and leakage ions in the neuron, respectively. *I*_*i*_, *I*_*k*_ (the total of *I*_*kv*_ and *I*_*kc*_), and *I*_*l*_ are corresponding currents of voltage sources. Note that *I*_*i*_ has a reverse direction of *I*_*k*_ and *I*_*l*_

Na/K-ATPase pumps consume ATP in order to create and maintain the concentration gradients of ions across the neuronal membrane, and therefore provide reversal potentials for each ion to generate electrical signals (Hammond 2008; Skou and Esmann 1992; Ogawa et al. 2009). In fact, Na/K-ATPase pumps store energy in the form of ion concentration gradients firstly during the refractory period, whereas the subsequent opening of ion channels rapidly dissipates this stored energy during relatively brief electrical signaling events, such as action potentials or spikes of bursting. More specificly, the biological energy ATP consumed by Na/K-ATPase pumps is transformed into the energy in the form of transmembrane concentration gradients of all the ions, which are the reversal potentials (*V*_*i*_, *V*_*k*_, *V*_*l*_) of each ion. As the electrical properties of neurons can be described in terms of electrical circuits, these reversal potentials can be regarded as the voltage sources in the electrical circuit (Fig. 4). Thus, the electrical potential energy consumption of the voltage sources representing the reversal potentials in the circuit, i.e. the stored energy by Na/K-ATPase pumps, is approximately the same as the consumed biological energy ATP.

According to Fig. 4, we firstly derive the following formula for the calculation of the power of the electrical potential energy consumption of the voltage sources (*V*_*i*_, *V*_*k*_, *V*_*l*_) in the circuit, which is also equivalent to the average power of consuming the energy stored by Na/K-ATPase pumps according to the analysis above.

4 $$P = |I_{kv} V_{k} | + |I_{kc} V_{k} | + |I_{l} V_{l} | - |I_{i} V_{i} |$$

$$\begin{aligned} I_{i} & = g_{i} m_{\infty }^{3} h_{\infty } (V - V_{i} ) \\ I_{kv} & = g_{kv} n^{4} (V - V_{k} ) \\ I_{kc} & = g_{kc} \frac{C}{1 + C}(V - V_{k} ) \\ I_{l} & = g_{l} (V - V_{l} ) \\ \end{aligned}$$

Note here that, the fourth item in formula (4) is—|*I*_*i*_*V*_*i*_|. This is because, in the circuit as well as in the neuron, the Na^+^–Ca^2+^ current (*I*_*i*_) is inward while K^+^ and leakage ion currents (*I*_*k*_ and *I*_*l*_) are outward. When these two inward and outward currents occur at the same time, the overlapping part of the electrical charges offset each other; therefore, it is their net power which contributes to the electrical potential energy (more properly, the membrane potential, such as resting potential and action potential) across the membrane. Thus, the ATP consumed by the overlapping current intrinsically determined by individual neurons, i.e. the neural models, is not contained in P here.

Throughout burst firing, the membrane potential is always below − 15 mV (Fig. 1), and the ion environment across the membrane remains positive outside and negative inside (Fig. 4). During a spike, the Na^+^–Ca^2+^ ion inflow (*I*_*i*_) releases the energy stored in the form of Na^+^ and Ca^2+^ ion concentration gradients. The outflow of K^+^ and leakage ions (*I*_*k*_ and *I*_*l*_) releases the energy stored in the form of K^+^ and leakage ion concentration gradients. Therefore, if P is positive at a certain moment, this means that the neuron’s consumption of stored energy in the form of K^+^ and leakage ion concentration gradients is greater than its consumption of stored energy in the form of Na^+^ and Ca^2+^ ion concentration gradients. Conversely, if P is negative, then the consumption of stored energy in the form of Na^+^ and Ca^2+^ ion concentration gradients outweighs the consumption of stored energy in the form of K+ and leakage ion concentration gradients. To simplify, we call positive P a net absorption of electrical potential energy and negative P a net release of electrical potential energy. This is because the outflow of *I*_*k*_ and *I*_*l*_ increases the electrical potential energy across the membrane while the inflow of *I*_*i*_ reduces it.

When P is positive, we write $P_{positive} = P\text{sgn} (P),(P \ge 0)$, then the net absorption of electrical potential energy in 30 s is $\int\limits_{0}^{T} {P_{positive} (t)dt} ,(T = 30\,{\text{s}})$. Conversely, when P is negative, we write $P_{negative} = P\text{sgn} (P),(P < 0)$, then the net release of electrical potential energy in 30 s is $\int\limits_{0}^{T} {P_{negative} (t)dt} ,(T = 30\,{\text{s}})$. During the generation of electrical signals, these two types of consumed energy derive from ion concentration gradients (Na^+^–Ca^2+^, K^+^ and leakage ions) stored by Na/K-ATPase pumps through the consumption of ATP before the outset of each electrical signal. During 30 s of bursting, the total of these two types of energy is therefore roughly equal to the ATP consumption of Na/K-ATPase pumps. We will call this total energy consumption “*E*”:

5 $$E = \int\limits_{0}^{T} {P_{positive} (t)dt + \int\limits_{0}^{T} {P_{negative} (t)dt} } ,(T = 30\,{\text{s}})$$

## Results

### No stimulus current

**Fig. 5:**
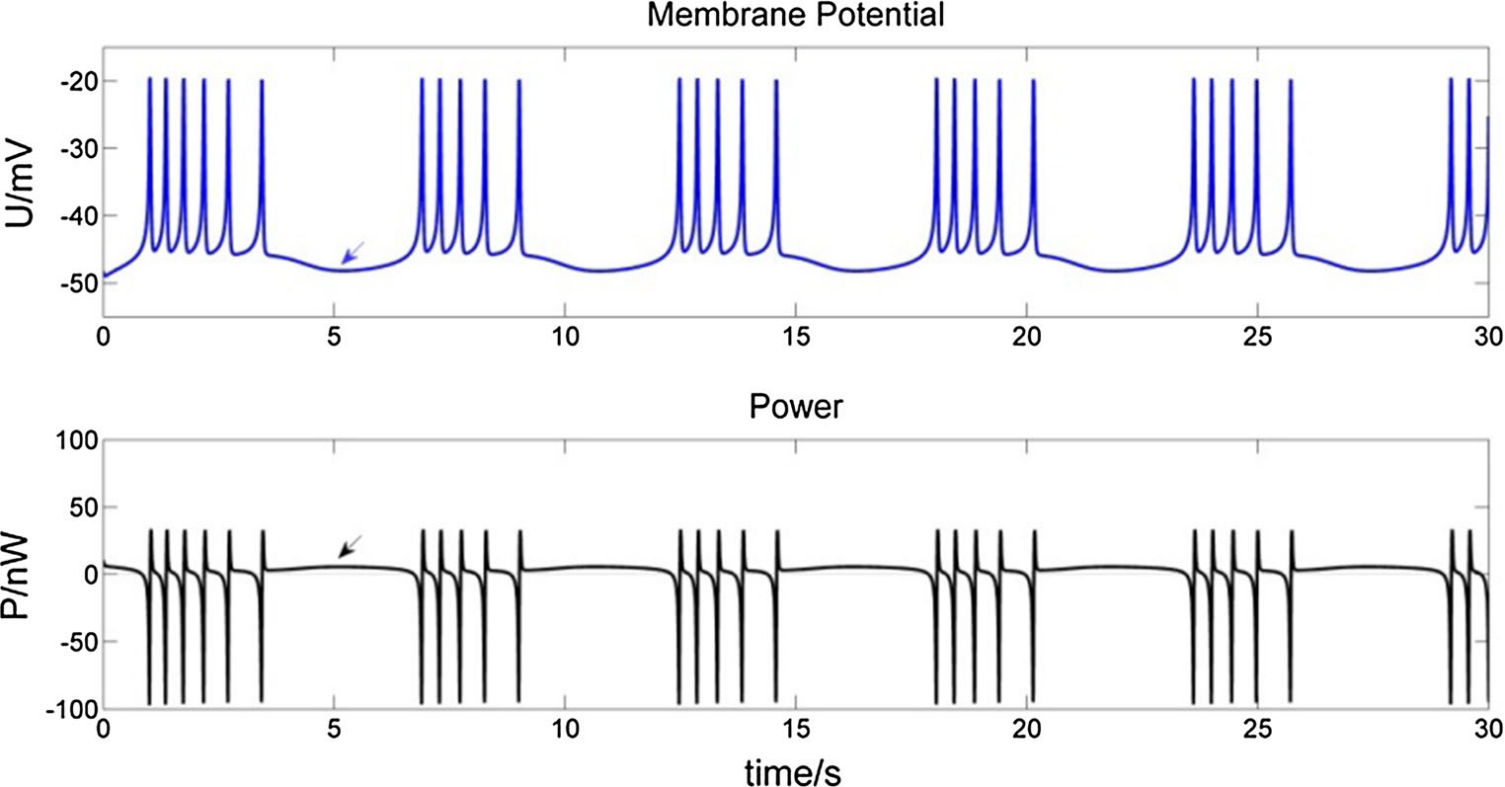
Membrane potential of a bursting neuron with *I *= 0 nA (top), and its corresponding power curve (bottom). (Top) The spikes in each burst undergo just two stages of depolarization and repolarization. Only after the final spike of a burst does the membrane potential exhibit hyperpolarization (top arrows), at which time the power value P approaches 0 nW (bottom arrows). (Bottom) During each spike, the corresponding P experiences alternating phases of electrical potential energy releasing (P < 0) and absorbing (P ≥ 0). Between bursts the neuron requires a significantly longer energy absorbing phase in order to recover resting potential

Figure 5 shows the membrane potential curve of a bursting neuron with stimulus current *I *= 0 nA and the corresponding power curve.

**Fig. 6:**
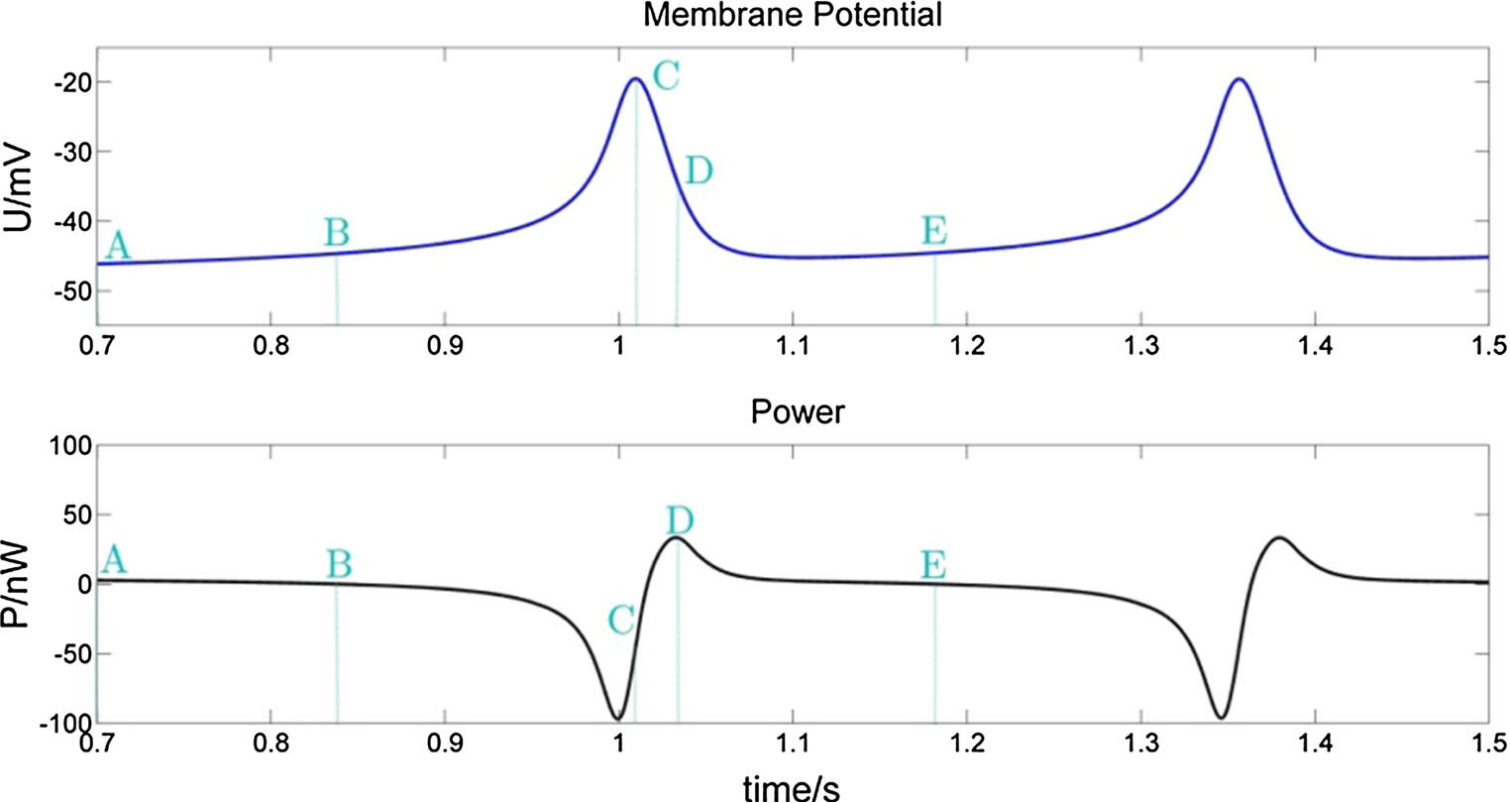
Enlarged section of the 0.7–1.5 s interval in Fig. 5. AB (resting potential), BC (depolarization), CD (early repolarization) and DE (late repolarization). A cycle for a spike of burst comprises BC, CD and DE. In AB, P is greater than but close to 0 nW. In BC, P remains negative starting from point B. The membrane potential reaches its maximum at C, while P reaches its maximum at point D. In DE, P declines to 0 nW at E

**Fig. 7:**
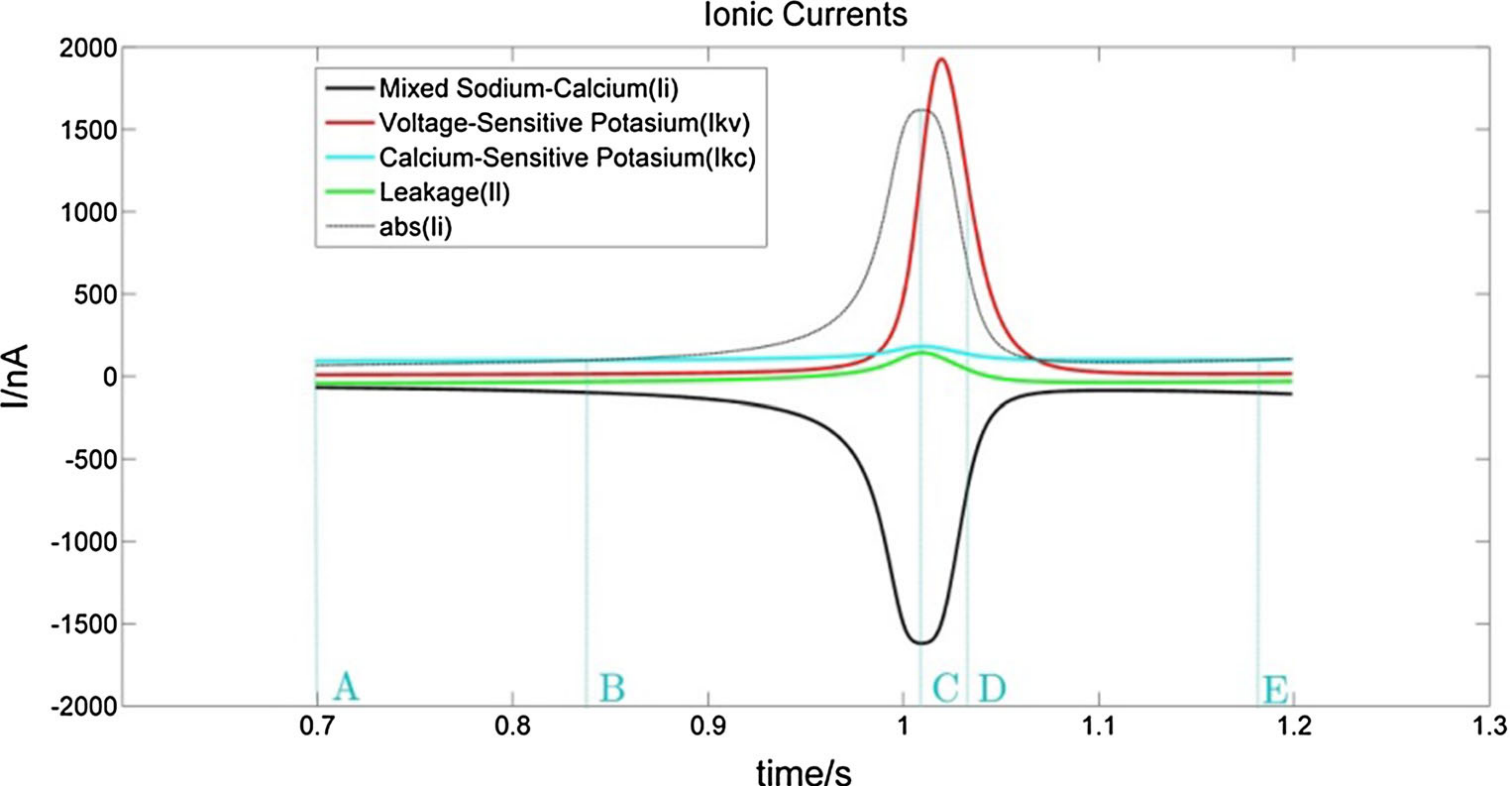
Ion currents in 0.7–1.2 s interval. The dark black line represents the Na^+^–Ca^2+^ mixed channel current, which is negative and in the direction of introversion. The voltage-gated Na^+^–Ca^2+^ mixed channel opens when the membrane potential reaches about − 45 mV and allows Na^+^ and Ca^2+^ ions to flow into the neuron. The light black line is the absolute value curve of the mixed Na^+^–Ca^2+^ ions current. The red line indicates the voltage-sensitive K^+^ channel current, which is positive and in the direction of extroversion. When the membrane is depolarized, the voltage-sensitive K^+^ channel opens and allows K^+^ ions to flow out of the neuron. The light blue line represents the Ca^2+^-sensitive K^+^ current with a positive value and in the direction of introversion. As the intracellular Ca^2+^ concentration increases, the Ca^2+^-sensitive K^+^ channel opens and allows K^+^ ions to flow out. Green line represents the other leakage current. (Color figure online)

In Fig. 6 below, we enlarge the 0.7–1.5 s interval in Fig. 5 and divide the first complete spike into 4 periods, which are AB (resting potential stage), BC (depolarization stage), CD (anterior segment of repolarization), and DE (posterior segment of repolarization). We now analyze one by one the change of membrane potential and its corresponding power for each segment. Figure 7 shows the variation curve of the ion current corresponding to Fig. 6, wherein the points A, B, C, D, and E correspond to each point in Fig. 6.

1. AB segment—resting potential stage

The neuronal membrane potential is about − 48 mV. Its corresponding power value P is greater than but close to 0 nW. As there are many more K^+^ channels without gate control than Na^+^ channels, the membrane permeability of K^+^ ions is higher than that of Na^+^ ions. Thus, even at resting potential, there will always be a small net outflow of K^+^ ions, which is consistent with the calculation that power value P is close to 0 nW.

(2) BC segment—depolarization stage

*Membrane potential change* When the membrane potential reaches − 45 mV, voltage-gated Na^+^–Ca^2+^ mixed channels open, which allows Na^+^ and Ca^2+^ ions to enter into the neuron and depolarize it. This process, in turn, results in more voltage-gated Na^+^ channels opening and further promotes membrane depolarization. The process continues and reinforces itself until the number of opening Na^+^ channels reaches a maximum and thus the membrane potential also reaches a maximum. In the BC segment, the voltage-sensitive K^+^ channels also open gradually, increasing the outward K^+^ current, which, however, remains much less than the inward Na^+^–Ca^2+^ mixed current, so that the membrane potential of BC segment rises until it reaches a maximum.

*Power change* Starting from point B, the power value P turns negative, gradually declining at first and then falling sharply to a minimum, after which it rebounds sharply. According to our definition of P, a negative value means that the net effect of the neuron is to release the energy stored in the form of Na^+^ and Ca^2+^ ion concentration gradients. This result is consistent with the previous research result in Zheng et al. (2014) that neurons first release stored energy very rapidly during the generation of action potentials.

In fact, during the early BC stage, it is the influx of Na^+^ and Ca^2+^ ions which mainly contributes to an increase of membrane potential and a decrease of the value P. During the later BC stage, after a short delay, the membrane depolarization reaches a level which triggers an opening of K^+^ channels and a large increase in the outflow K^+^ ions. However, the Na^+^–Ca^2+^ ions are still greater than the outflow of K^+^ ions, so that the membrane potential continues to rise until the inward current of Na^+^ and Ca^2+^ ions roughly equals the outward currents of K^+^ ions and leakage ions (see Fig. 7). It is worth noting that in the later stage of the BC phase, the power P, after reaching its minimum value, begins to rise again, because the voltage-sensitive K^+^ current, though smaller than the mixed Na^+^–Ca^2+^ current, rises faster.

(3) CD segment—repolarization early phase

*Membrane potential change* When the membrane potential reaches its maximum at C point, the inward and the outward ion flows are basically the same. As the high permeability of Na^+^ ions lasts only for a short time, the inflow of Na^+^ ions decreases, while the permeability of K^+^ ions increases sharply. Since the outward voltage-sensitive K^+^ current is much greater than the inward Na^+^–Ca^2+^ mixed ion current, the membrane potential repolarizes quickly. During this phase, the outward voltage-sensitive K^+^ current increases to a maximum then decreases rapidly, but remains larger than the inward Na^+^–Ca^2+^ ion current, resulting in a downward trend in membrane potential.

*Power change* As the outward voltage-sensitive K^+^ current is much larger than the inward Na^+^–Ca^2+^ mixed current after the C point, the power value P begins to rise and turns positive. With more and more K^+^ ions flowing out, P increases until the outward ion current (K^+^ and leakage current) is larger than the inward ion current (Na^+^–Ca^2+^ mixed current). P continues to rise, reaching its maximum at point D.

Even though the membrane potential reaches its maximum at C, the inward ion flow remains slightly larger than the outward ion flow (see Table 1). Therefore the power value P at this point is still negative. After a very short period of 0.006 s, P rises to 0 nW.

**Table 1:** Values of ion currents at C point

Ionic currents	*I*_Na–Ca_ (nA)	*I*_KV_ (nA)	*I*_KC_ (nA)	*I*_L_ (nA)
C point	−1619	1249	182.7	143.7
		1575.4

(4) DE segment—repolarization late phase

After point D, the membrane permeability for K^+^ and for the Na^+^–Ca^2+^ mixed ions both continue to decline. Thus, the membrane potential decreases, returning to the resting potential. The power value P declines to 0 with the decrease of the K^+^ ion outflow. At the same time, the outward (two K^+^ currents) and the inward (Na^+^–Ca^2+^ mixed current and leakage current) ion flows remain almost equal. These four stages are repeated in the subsequent spikes in neuronal bursting.

The above analysis describes the ionic mechanism of the change of membrane potential and its corresponding power for each spike during bursting. The spikes in each burst undergo just two stages of depolarization and repolarization. Only after the final spike of a burst does the membrane potential exhibit hyperpolarization (Fig. 5 top arrows), at which time the power value P approaches 0 nW (Fig. 5 bottom arrows). This phenomenon is different from action potential (AP), which exhibits hyperpolarization in each spike, due to the overriding effect of outward K^+^ currents, which outweigh the effect of inward Na^+^–Ca^2+^ and leakage currents. In Fig. 5, we also can observe that the bursting neuron experiences alternating phases of electrical potential energy release and absorption. Between bursts the neuron requires a significantly longer energy absorbing phase in order to recover resting potential.

### Different stimulus currents

**Fig. 8:**
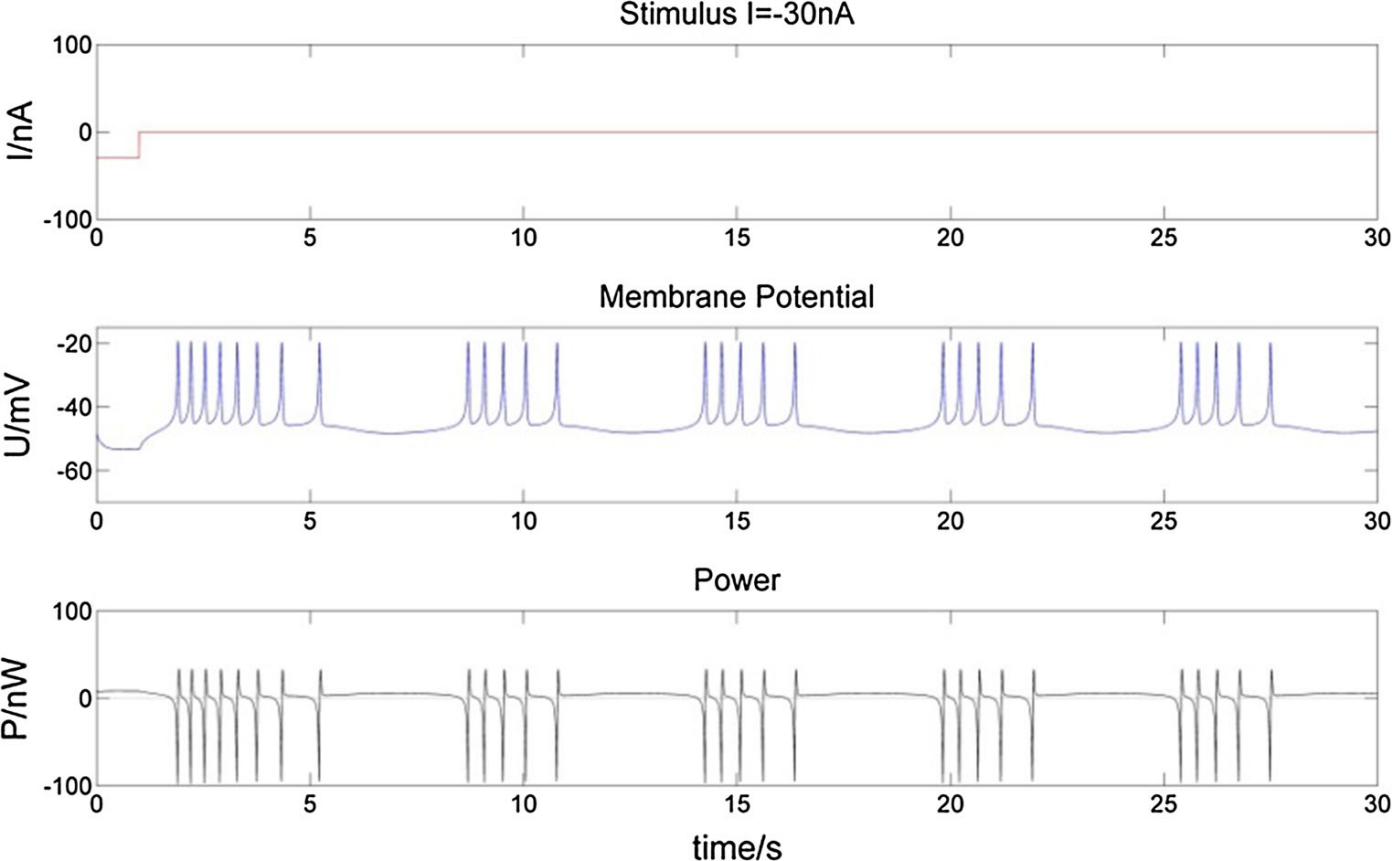
Membrane potential of a bursting neuron and its power change, from 0 to 1 s stimulated by − 30 nA current. The neuron hyperpolarizes during the 1 s negative stimulus while P increases compared to no stimulus in Fig. 5. Then the membrane potential rises to fire spikes. The number of spikes in the first burst increases with the frequency slows down throughout the burst. The negative stimulus has no effect on subsequent bursts

**Fig. 9:**
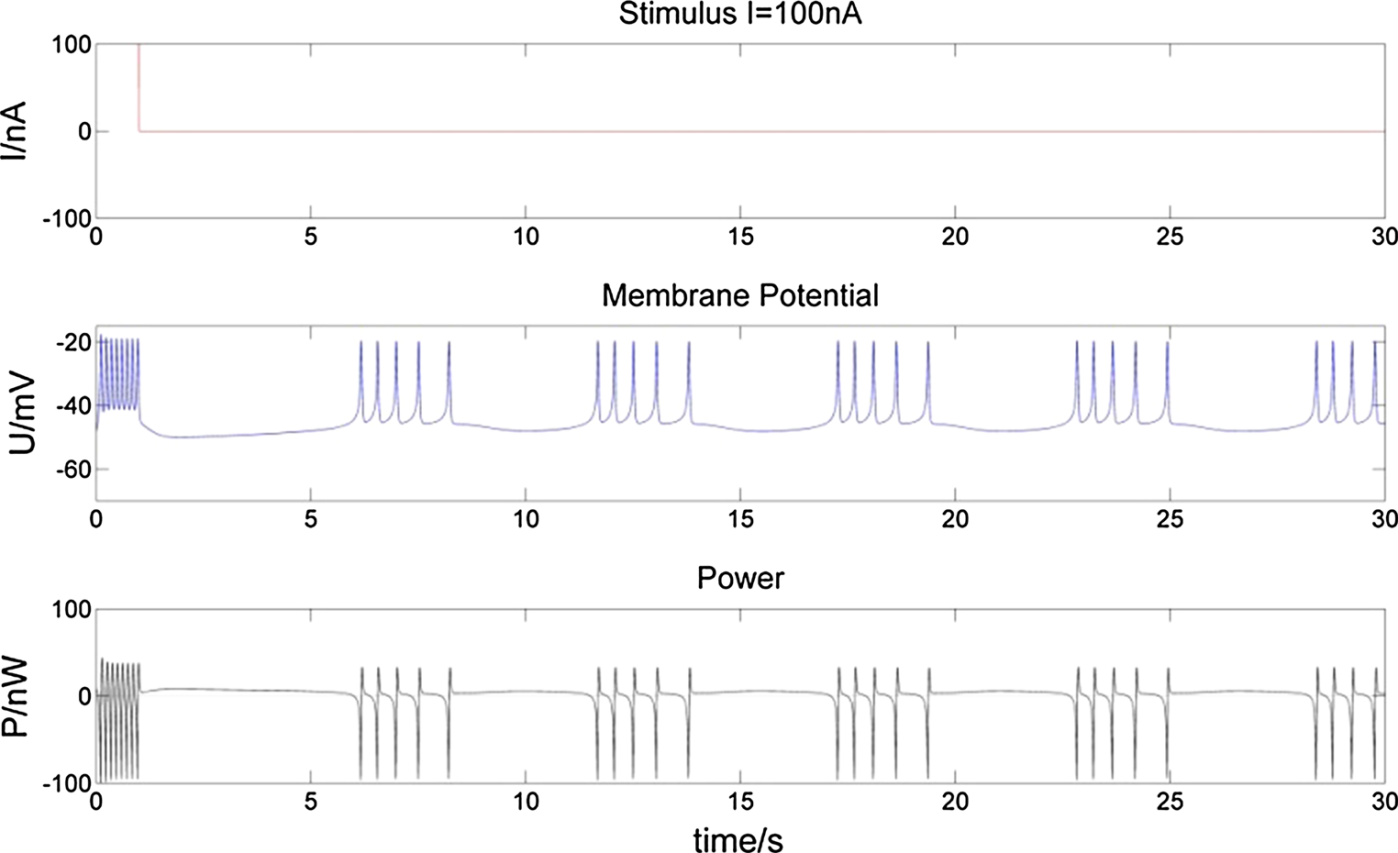
Membrane potential of a bursting neuron and its power change, from 0 to 1 s stimulated by 100 nA current. The positive stimulus depolarizes and repolarizes the membrane potential rapidly. The number of spikes in the first burst increases as well as their frequency. There is a longer hyperpolarization and incremental P after the first burst, but no change to following bursts

**Fig. 10:**
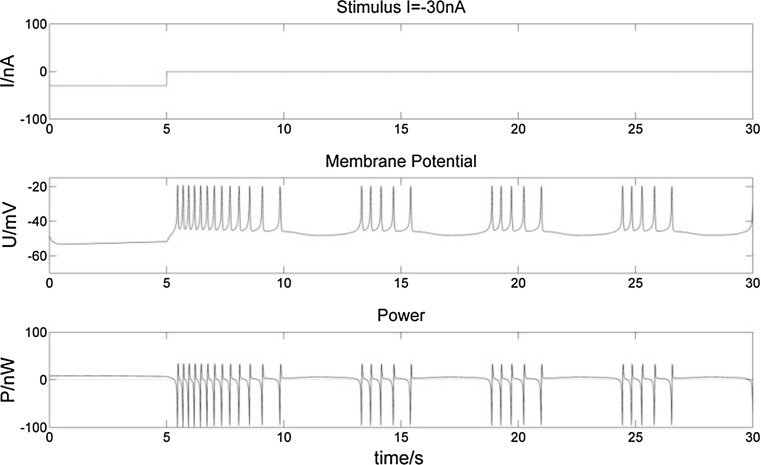
Membrane potential of a bursting neuron and its power change, from 0 to 5 s stimulated by − 30 nA current. Compared to Fig. 8, the longer negative stimulus gives rise to a longer hyperpolarization of the neuron. The number of spikes in the first burst increases more, while there is still no influence on subsequent bursts

**Fig. 11:**
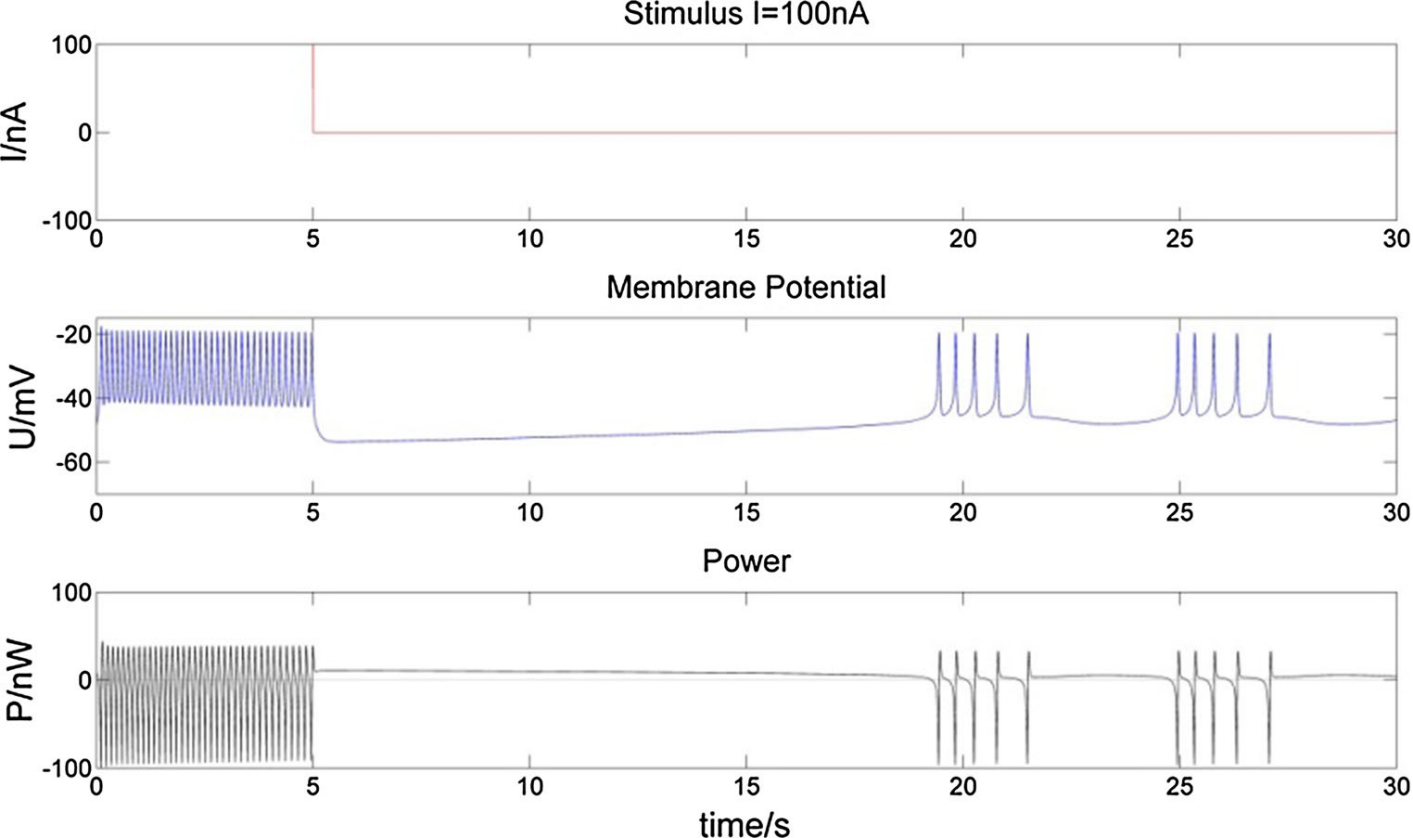
Membrane potential of a bursting neuron and its power change, from 0 to 5 s stimulated by 100 nA current. Compared to Fig. 9, the increasing number of spikes in the first burst lasts longer with the longer positive stimulus. There is a much longer hyperpolarization period and corresponding incremental P after the first burst

**Fig. 12:**
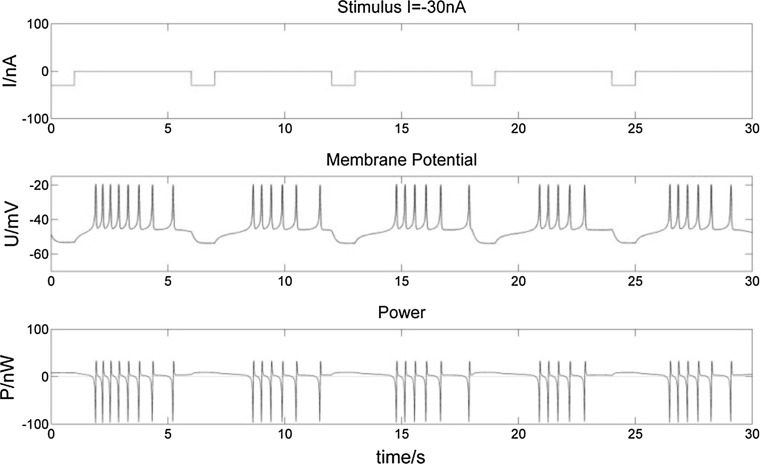
Membrane potential of a bursting neuron and its power change with an intermittent − 30 nA stimulation of 1 s in every 5 s. The neuron hyperpolarizes more intermittently when stimulated. Bursts are influenced by the stimuli as well

**Fig. 13:**
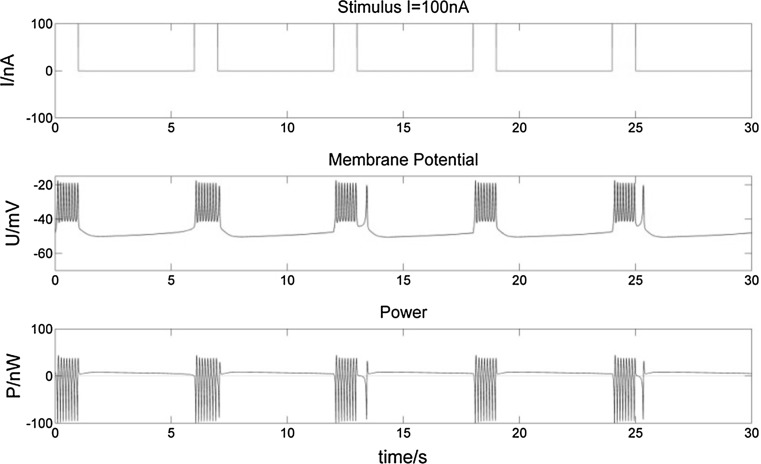
Membrane potential of a bursting neuron and its power change with an intermittent 100 nA stimulation of 1 s in every 5 s. Bursts of higher-frequency spikes are synchronous with positive stimuli, as well as P value

To study the various power changes when a bursting neuron receives a stimulus, we consider three types of stimulus inputs: (1) a one-second (1 s) stimulus (Figs. 8, 9), a 5 s stimulus (Figs. 10, 11), and intermittent stimuli of 1 s every 5 s (Figs. 12, 13).

From the membrane potential changes of the bursting neuron illustrated in Figs. 8, 9, 10, 11, 12 and 13, three results can be observed: (1) A positive stimulus of 0–1 s increases the number of spikes of bursts and causes longer hyperpolarization after the first burst, while a stimulus of 0–5 s has a similar but greater effect. In both cases there is no effect on the following bursts. (2) A negative stimulus of 1 s or 5 s makes the neuron prone to hyperpolarization. The greater the current value and the longer the stimulus, the more obvious the hyperpolarization phenomenon is; but a longer stimulus has no influence on subsequent bursts. (3) For intermittent stimuli, the effects of positive and negative currents on the bursting neuron are similar to those of a single positive and negative 1 s and 5 s stimulus, respectively, except that positive stimuli produce synchronous bursts (Figs. 7, 8, 9).

Stimulation modifies the ion currents crossing the membrane. Firstly, a positive current (Figs. 9, 11, 13) lowers the negativity of the intracellular environment, allowing the Na^+^–Ca^2+^ ion channel to open faster, which in turn accelerates the opening of voltage-sensitive K^+^ ion channels. As a result, the membrane potential is depolarized and repolarized faster than without stimulation. In addition, after each spike, outward K^+^ and inward Na^+^–Ca^2+^ ion currents reach a balance earlier. As the Ca^2+^-sensitive K^+^ ion current is still greater, the membrane potential is more likely to rise to cause another spike. At the repolarization phase of the final spike, the voltage-sensitive K^+^ ion current is greater than the Na^+^–Ca^2+^ ion current, which makes the membrane potential more negative than without stimulation during hyperpolarization. Since more Ca^2+^-sensitive K^+^ ions must flow out of the neuron, more time is needed to reach the resting potential. Secondly, when stimulated by a negative current (Figs. 8, 10, 12), the intracellular environment becomes more negative, which delays the opening of the Na^+^–Ca^2+^ ion channel. It takes longer to reach the threshold − 45 mV for depolarization. In general, positive stimulus currents give rise to higher bursting frequencies with longer and more pronounced hyperpolarization, while negative currents inhibit neuronal firing.

### Total energy consumption in 30 s

#### No stimulus current input

According to formula (5), when *I *= 0 nA, the total energy consumed by a single neuron during 30 s of bursting is 215.2 nJ = 2.152 × 10^−7^ J (the second row in Table 2). This is very similar to the result of the biological energy supplied by ATP of 2.468 × 10^−7^ J during the generation of a single action potential (Wang et al. 2017a). It is obvious that the neuron’s energy consumption is very low during spontaneous bursting activity.

**Table 2:** The total energy consumed by a bursting neuron with different stimulus current inputs

Stimuli	*I* (nA)	Total energy consumption (nJ)
0–1 s	0	215.2010
	− 30	218.7014
	40	228.9818
	100	235.3603
0–5 s	− 30	233.7486
	40	288.7737
	100	335.8633
Intermittent	− 30	240.6388
	40	286.6957
	100	320.4553

These stimuli listed in this table also correspond to those in Figs. 8, 9, 10, 11, 12 and 13

#### Different stimulus current inputs

**Fig. 14:**
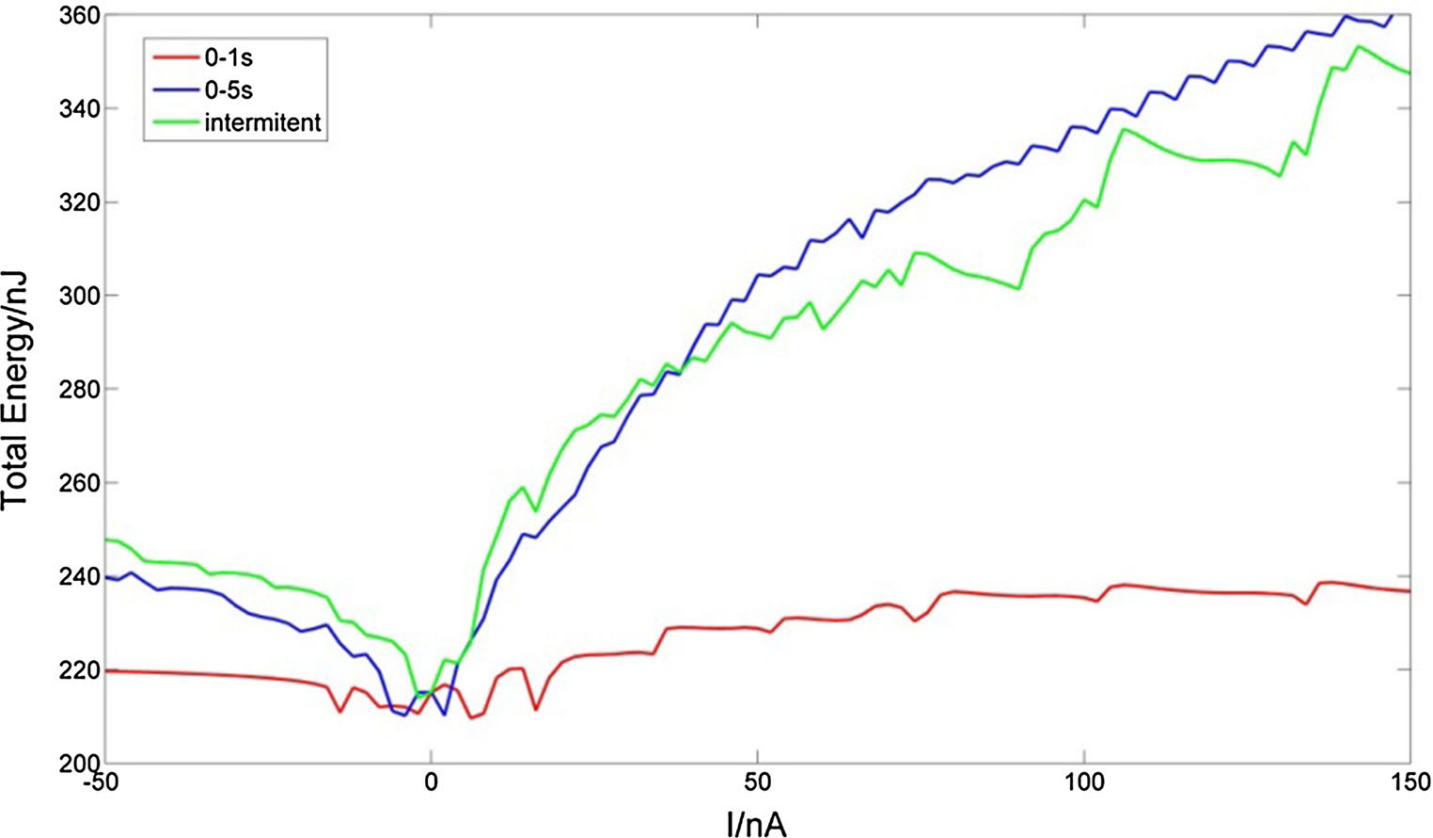
Changes in total energy consumption during 30 s of bursting with various types of stimuli. When stimulated for 0–1 s, the neuron’s total energy consumption remains low (209.6130–238.6591 nJ) regardless of the magnitude of the current stimulus (red line). By contrast, when stimulated during 0–5 s, total energy consumption varies greatly (210.1846–361.7620 nJ) (blue line). When intermittent stimulation is applied, total energy consumption is similar to that of 0–5 s (213.9738–353.3333 nJ) (green line). (Color figure online)

In Fig. 14 we show change in total energy consumption when the neuron is subjected to the above mentioned stimuli: 0–1 s, 0–5 s and intermittent 1 s every 5 s, with currents ranging in value from − 50 to 150 nA.

In Fig. 14, with the same intensity, the longer the stimulus, the more energy is consumed by the bursting neuron, as shown by the red and blue lines. The similar shape of the blue and green lines shows that when the total duration of the stimulus is the same, the total energy consumption is also fundamentally the same even though the types of stimulus are different.

Moreover, Fig. 14 also shows the neuron’s total energy consumption is low with stimuli near 0 nA. For both positive and negative current stimulus, the total energy consumed increases as the absolute value of the current increases. This phenomenon is consistent with the brain’s mechanism of maximization of energy utilization (Zheng and Wang 2012; Laughlin and Sejnowski 2003). In fact, when the reception of information from other neurons increases, i.e. when the electrical stimulus increases, the energy consumed by the neuron increases, and vice versa.

## Conclusion and discussion

Based on the Chay model, this paper proposes a method to calculate neural energy consumption according to the effect of ion flows on the membrane potential during subthreshold firing activities of a bursting neuron. We first calculate the neural energy transferred in a unit of time, i.e. the power. The total energy consumption of the bursting neuron in 30 s is calculated according to the energy conservation law. We analyze the changes of the membrane potential and neural energy consumption in detail and come to the following conclusions about neuronal bursting activities:

1. Each burst begins with a phase of resting potential, during which the energy stored in the form of K^+^ ion concentration gradient consumed and transferred to the electrical potential energy across the membrane. Then come a number of spikes in which the energy is first released from Na^+^ and Ca^2+^ ion concentration gradients (the power value becomes negative) during depolarization, then the electrical potential energy absorbs again the energy stored in the form of K+ and leakage ion concentration gradients during repolarization. At the end of this sequence comes a final phase of hyperpolarization during which more electrical potential energy is collected, from the effect of absorption of the energy from K^+^ and leakage ion concentration gradients, to return the resting potential before the next burst.
2. A positive electric stimulus triggers more spikes during a burst and a longer phase of hyperpolarization after the last spike. A longer positive stimulus triggers even more spikes, resulting in a longer final hyperpolarization phase to absorb more energy from ion concentration gradients in order to return to the resting potential. A negative stimulus, by contrast, inhibits spikes.
3. During bursting activities, the neuron’s total energy consumption in 30 s is low. In the absence of stimulus, the energy consumption of the bursting neuron remains near the minimum. In fact, the total energy consumed (2.152 × 10^−7^ J) during 30 s of bursting is very similar to the biological energy supplied by ATP of 2.468 × 10^−7^ J during a single action potential. With a positive stimulus, energy consumption increases. And the greater the stimulus, the larger the increase in energy consumption, regardless of the length of the stimulus from 1 to 5 s. The effect of a negative stimulus is similar, with energy consumption increasing in line with the absolute value. If a stronger stimulus can be equated to the transfer of more information, the brain consumes more energy in processing the information. All these demonstrate how efficiently the brain uses energy.

These conclusions are in line with the previous results (Zheng and Wang 2012; Zheng et al. 2014; Wang et al. 2017a), indicating that the neural energy calculation method proposed in this paper is reasonable. This can provide a theoretical basis for understanding the dynamics of subthreshold neural activity and contribute to decrypting neural coding. Moreover, since neural energy has the property of additivity (Wang et al. 2015b), it can be assumed that the methods developed in this paper can be transferred from the level of the single neuron to neural networks. Our goal is to examine the quantitative relationship between neural signals which carry information and neural energy consumption. The results of this research may 1 day open the door to a better understanding of the coding mechanisms and the functioning of the brain.
